# Fabrication of Terahertz Fresnel Zone Plates via Ultraprecision Mechanical Processing

**DOI:** 10.3390/mi17030368

**Published:** 2026-03-19

**Authors:** Meng Chen, Jinshi Wang, Fengzhou Fang

**Affiliations:** State Key Laboratory of Precision Measurement Technology & Instruments, Tianjin University, Tianjin 300072, China; chenmeng456@tju.edu.cn

**Keywords:** Fresnel zone plates, ultraprecision machining, terahertz

## Abstract

This study proposes a new fabrication process for terahertz Fresnel zone plates on high-resistivity silicon substrates. It involves ion implantation surface modification, ultra-precision diamond turning, and magnetron sputtering, followed by polishing. Ductile-regime cutting is used to form smooth microgrooves, which are selectively metallized to create alternating opaque and transparent zones for terahertz waves. Finite-element simulations are performed to design the zone structure and to evaluate the effect of process-induced radius errors. A 3 μm amorphous layer is formed via ion implantation, which significantly enhances the ductile-to-brittle transition depth of silicon from 55 nm to about 535 nm while causing only minor changes in terahertz transmittance. The results demonstrate that the proposed method can produce high-quality Fresnel zone plates on silicon and offers a practical route to compact diffractive terahertz components.

## 1. Introduction

Terahertz (THz) waves refer to electromagnetic radiation in the frequency range of 0.1–10 THz. Owing to their strong penetration, THz waves are widely applied in communications [1], biomedicine [2], agriculture [3], and security screening [4]. THz focusing is essential for achieving high-resolution imaging and precise inspection. Terahertz refractive lenses, such as plano-convex and aspheric lenses, typically have a center thickness of several centimeters. For instance, a PTFE plano-convex lens with a diameter of about 100 mm can have a center thickness of 30 mm, and an f-theta aspheric lens for scanning systems can exceed 40 mm in thickness, which limits their use in lightweight and miniaturized systems. In contrast, a Fresnel zone plate (FZP) is a typical diffractive focusing element [5] that offers a compact form factor. THz FZPs on various materials have been developed. For organic materials (e.g., polyamide), 3D printing enables rapid prototyping of THz FZPs; for example, Furlan et al. [6] fabricated a phase-type FZP operating at 0.625 THz for THz focusing and imaging systems. However, the limited structural resolution hampers the fabrication of narrower zone widths, thereby restricting the maximum number of zones. For crystalline materials (e.g., Si and GaAs), fabrication typically relies on laser direct writing or semiconductor microfabrication processes. For example, Indrišiūnas and Minkevičius et al. [7,8] produced a 32-level phase FZP using femtosecond-laser direct writing, with experiments demonstrating that increasing the phase-quantization level effectively improves focusing efficiency. Nonetheless, surface damage caused by laser ablation can reduce THz transmission efficiency, even when the roughness within the processed area is much smaller than the operating wavelength. Al-Daffaie et al. [9] fabricated a Fresnel lens antenna with high-aspect-ratio structures via anisotropic wet etching, which can serve as an alternative to conventional convex lenses. However, limited process control leads to micrometer-scale deviations in the dimensions of the microstructure.

Ultra-precision diamond turning (UPDT) is an effective method for creating micro- and nanostructures on optical-crystal surfaces, and it can achieve nanometer-level form accuracy for spherical, aspheric, and freeform geometries [10,11,12]. It is especially well-suited for fabricating rotationally symmetric structures such as FZPs. High-resistivity silicon (HR-Si) is an ideal material for terahertz applications, and its cutting mechanisms and process development have been studied extensively. In practice, enabling the brittle-to-ductile transition and sustaining stable ductile-regime material removal are key to damage-free machining of brittle materials such as silicon. Leung et al. [13] achieved a surface roughness Sa of 2.86 nm by controlling machining parameters. They suggested that ductile-regime cutting requires the undeformed chip thickness to remain below a critical value to obtain high-quality nanometric surfaces. Fang et al. [14] proposed that, during nanometric cutting, material removal is governed primarily by extrusion rather than shearing. Based on this, they conducted cutting experiments on single-crystal silicon and obtained a smooth surface of 1 nm in Sa. Yan et al. [15] found that the thickness of the subsurface amorphous layer is highly sensitive to the tool rake angle and cutting depth, and they proposed a subsurface damage model. Because crystalline materials are both hard and brittle, surface fracture remains a major challenge, as it increases scattering and degrades optical quality. To mitigate surface microcracks, an ion implantation surface modification technique has been developed to reduce surface hardness and brittleness, thereby significantly improving machinability [16]. This method employs energetic particle-beam bombardment to induce damage in the surface lattice; as the implantation dose increases, defects accumulate, leading to surface amorphization and the formation of a more machinable layer. This approach has been validated for hard, brittle materials such as Si [17], Ge [18], and SiC [19]. For example, implanting F ions into single-crystal silicon at 10 MeV with a dose of 1 × 10^14^ cm^−2^ increased the ductile-to-brittle transition depth (DBTD) from 236 nm to 923.566 nm [16]. Similarly, Cu-ion implantation in single-crystal germanium at 3 MeV with a dose of 1 × 10^16^ cm^−2^ increased the measured brittle-to-ductile transition depth (BDTD) from 55 nm to a maximum of 730 nm [18]. Molecular dynamics simulations have further clarified the atomic-scale mechanisms and the effects of implantation dose and cutting parameters [20,21,22]. In contrast, studies have shown that if ion implantation does not fully amorphize the material, the modification may be weakened or even become detrimental [23]. Experimental results indicate that non-amorphizing implantation significantly alters the anisotropic cutting behavior of silicon, reducing the undeformed chip thickness by 41% along the <100> direction. To reduce the high dose required to form a micrometer-scale amorphous layer, a multi-implantation surface modification strategy has been proposed [24]. Compared with single implantation, this method can reduce the total implantation dose by 80% without sacrificing the modification quality, thereby significantly lowering process costs. In addition, optimizing the implantation energy and sequence helps produce a more uniform modified layer. This strategy has enabled crack-free cutting of silicon micropillar arrays [24] and microlens arrays on germanium substrates [25].

This study develops an integrated process chain for fabricating THz FZPs on HR-Si based on ultra-precision machining. In this route, ultra-precision diamond turning (UPDT) defines the diffractive microgroove geometry. At the same time, ion implantation is incorporated as an enabling step to modify the near-surface layer, improve machinability, and suppress brittle-fracture damage during groove formation. Magnetron sputtering followed by polishing is then employed to selectively retain metal within the microgrooves, thereby forming the alternating opaque and transparent annular zones required for THz FZPs. The experiments verify that the proposed process chain can reliably produce high-quality THz FZPs, achieving a zone-radius error within 5 μm and a surface roughness below 2 nm.

## 2. Materials and Methods

### 2.1. Design of THz FZP

Fresnel zone plates (FZPs) can be classified into amplitude and phase types. Because terahertz wavelengths are about hundreds of micrometers, the structural depth required for phase-type zone plates becomes impractically large. Therefore, this study focuses on the amplitude type. The FZP consists of concentric rings with alternating transparent and opaque zones. Waves transmitted through the FZP arrive in phase at the designated focal point, resulting in constructive interference and enhanced focal intensity. In contrast, the opaque zones block the waves that would otherwise cause destructive interference at the focal point. The zone radius Rn can be calculated using Equation (1) [5]:

(1) $$R_{n}=\sqrt{n\lambda F+{\left(n\lambda /2\right)}^{2}}$$

where *n* is a positive integer sequence, *λ* is the operating wavelength, and *F* is the focal length.

A two-dimensional axisymmetric FEM model of the FZP is built in COMSOL Multiphysics 5.5 (COMSOL, Inc., Burlington, MA, USA). to further examine the details of the electric-field distribution. The operating frequency of the FZP is set to 1 THz, and the focal length to 20 mm. The thickness of the substrate is 1 mm, and the functional layer consists of coplanar transparent and opaque annular zones with a uniform thickness. Based on the electric displacement vector analysis, HR-Si is assigned to the transparent annular zones with a relative permittivity of ε_r_ = 11.9 and a conductivity σ ≈ 0 at 1 THz. The opaque blocking zones are composed of metallic aluminum (Al), with a conductivity of 3.77 × 10^7^ S/m. The incident electric-field amplitude is set to 1 V/m, and all other boundaries are terminated with perfectly matched layers (PMLs) to absorb outgoing waves and suppress reflections. In the PML region, the mesh is discretized using a quadrilateral grid with a minimum element size of 30 μm (i.e., *λ*/10). The substrate is meshed with triangular elements, with a minimum element size of 15 μm (i.e., *λ*/20). The remaining propagation domain is meshed using triangular elements with a minimum size of 30 μm (i.e., *λ*/10). To investigate how the number of zones affects focusing performance, FZP models with 10, 20, and 30 zones are constructed, and their electromagnetic-field distributions, focal full width at half maximum (FWHM), and focal energy fraction are evaluated. To further assess the impact of fabrication errors, the width of each transparent zone is increased or decreased by 20%. Meanwhile, the width of the adjacent opaque zone is adjusted inversely (i.e., decreased or increased accordingly) to keep the radial position of each zone center unchanged relative to the FZP center.

### 2.2. Fabrication Process

**Figure 1:**
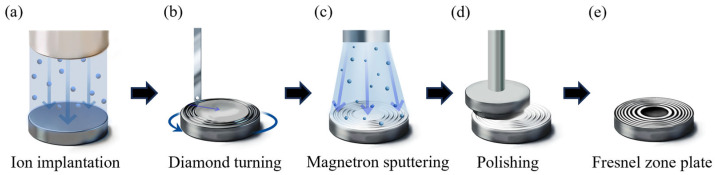
Fabrication process of the THz FZP in this study: (**a**) ion implantation surface modification; (**b**) ultra-precision diamond turning; (**c**) magnetron sputtering; (**d**) polishing; and (**e**) the fabricated Fresnel zone plate.

A single-crystal HR-Si substrate (double-sided polished, (111) orientation, 1000 Ω·cm) with a 30 mm diameter and 1 mm thickness is used. The fabrication process is shown in Figure 1. First, the workpiece is irradiated with high-energy ions to reduce the mechanical strength and improve machinability. UPDT is used to generate concentric annular grooves on the substrate, corresponding to the opaque rings of the FZP. Next, a metal layer is deposited via magnetron sputtering and subsequently polished to remove the film from the plateau regions while retaining metal within the microgrooves. Finally, the FZP structure with alternating opaque and transparent zones is obtained.

Ion implantation parameters are determined using the Stopping and Range of Ions in Matter (SRIM) [26], with the aim of producing a 3 μm thick amorphous layer through multiple implantations. The ion species, energies, and doses for each step are listed in Table 1. The implantation energy is increased with the implantation number so that the shallow layer is preferentially modified. This sequence helps suppress channeling effects during subsequent higher-energy implantations and improves the uniformity of the modified layer. The experiments are performed at room temperature using a tandem electrostatic accelerator. The incident ion beam is tilted by 7° relative to the surface normal to prevent the channeling effect. During implantation, the beam current is maintained between 65 and 900 nA.

Diamond turning is conducted on a Moore 250 ultra-precision lathe (Moore Nanotechnology Systems, LLC, Keene, NH, USA). Considering the minimum mesh size used in the simulations, the target structural accuracy of the FZP is set to 15 μm. The microgroove depth is set to 2 μm to prevent removal of the metal film inside the grooves during subsequent polishing and to keep the cutting region within the modified layer. The modification depth is set to approximately 3 μm. Because the modified layer must cover both the depth of the groove and the surface tilt error introduced by fixturing and leveling, the structure depth is 2 μm, and the parallelism error is about 1 μm. The minimum groove width is 261 μm; therefore, a single-crystal diamond tool with a nose radius of 0.192 mm is chosen. The spindle speed is 1250 rpm, and the feed rates in the X and Z directions are 0.675 mm/min and 0.4 mm/min, respectively. The tool rake angle is −12°, and cutting fluid is used throughout machining. Because the amorphous-layer thickness is comparable to the groove depth, a dedicated fixture is designed to precisely tilt and align the workpiece, ensuring that the axial runout of the workpiece surface during spindle rotation stays within 1 μm.

After machining the Fresnel structure, the deposited metal film must meet two requirements. First, it should be thick enough to effectively absorb terahertz waves. Second, it should strongly adhere to the substrate to prevent delamination inside the microgrooves during polishing and to maintain the integrity of the opaque zone. Therefore, magnetron sputtering is used to deposit a 5 nm Cr adhesion layer on the silicon substrate, followed by a 300 nm Al film.

The polishing procedure aims to remove the metal film from the plateau regions outside the microgrooves, thereby creating the transparent zones of the FZP. A synthetic leather pad and an alumina slurry with a particle size of 50 nm are used. The samples are polished for 10 min under a pad pressure of 180 kPa and a rotation speed of 80 rpm. Under these conditions, the metal film could be removed without damaging the silicon surface, and the use of fine abrasive particles helps avoid damage to the metal layer retained inside the grooves.

### 2.3. Measurement and Characterization

A white-light interferometer (Wyko NT9100, Veeco Instruments Inc., Tucson, AZ, USA) in PSI mode is used to assess surface roughness. A contact profilometer (Form Talysurf PGI, Taylor Hobson Ltd., Leicester, UK) is used to measure the microstructure dimensions along the diameter after diamond turning. To investigate the change in material properties by ion implantation, Raman spectroscopy is performed using a modular Raman microscope (iHR320, HORIBA Scientific, Kyoto, Japan) equipped with a 532 nm laser and an 1800 grooves/mm grating, and nanoindentation was performed using a Berkovich indenter (Micro Star Technologies, Huntsville, TX, USA) with a maximum indentation depth of 2 μm and a strain rate of 0.05 s^−1^ to evaluate the hardness and elastic modulus. Surface morphology is examined using a scanning electron microscope. In addition, a custom-built transmission-mode terahertz time-domain spectroscopy (THz-TDS) is used to characterize the transmittance of the single-crystal and ion-implanted silicon samples. Measurements are performed with 1 mm thick samples positioned at the beam focus, using air as the reference. A point detector records the transmitted time-domain terahertz pulse, and the transmittance spectrum over 0.5–1.5 THz is obtained by Fourier-transforming the measured waveforms. The surface 3D profile is acquired using the laser mode (10× objective) of an Olympus confocal microscope (Olympus Corporation, Shinjuku, Tokyo).

## 3. Results and Discussion

### 3.1. Stimulation

**Figure 2:**
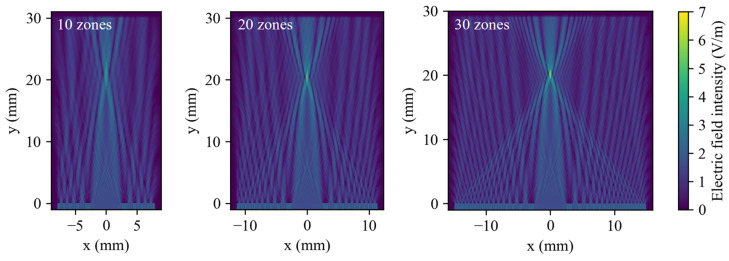
Electromagnetic field distributions of 10-, 20-, and 30-zone FZPs.

**Figure 3:**
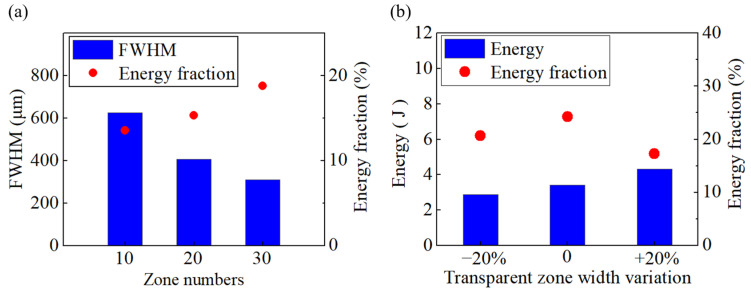
(**a**) Focal full width at half maximum (FWHM) and energy fraction within the focal spot. (**b**) Total energy on the focal plane and focal energy fraction for different transparent-zone widths.

For FZP models with 10, 20, and 30 zones, the corresponding electric field distributions are shown in Figure 2, and the FWHM and energy fraction are summarized in Figure 3a. The results indicate that, as the number of zones increases, both the peak intensity and the focal energy fraction increase, while out-of-focus sidelobes and background noise decrease, leading to a higher focusing contrast. At the same time, the focal spot diameter decreases, indicating that a larger effective aperture improves lateral resolution. Simulations with a ±20% variation in the widths of the transparent rings show that a well-defined focus still forms near the focal plane in all three cases. Figure 3b displays the energy along the focal line and the focal energy fraction. As the transparent-ring width increases, the total energy increases, and the energy at the focal plane increases accordingly; however, the highest focal energy fraction occurs for the error-free structure, indicating that the focusing performance of the FZP relies on structural accuracy. This is because constructive interference is most prominent in this case. Widening the transparent region causes more electromagnetic waves with non-constructive interference to be concentrated in the focal region, resulting in a decrease in the energy fraction at the focal point. In contrast, narrowing the transparent region reduces the transmitted energy but still maintains stricter phase-matching conditions. Therefore, the energy fraction in the focal region is higher for the narrowed transparent rings than for the widened transparent rings.

### 3.2. Surface Modification

**Figure 4:**
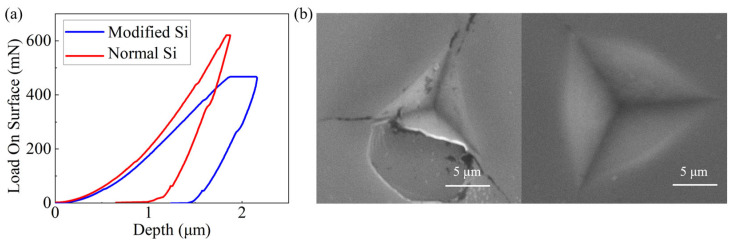
(**a**) Loading–unloading curves obtained from nano-indentation tests and (**b**) nano-indentation surface topology.

After ion implantation, the load–displacement curves (Figure 4a) demonstrate that, at the same indentation depth, the required load is significantly lower after modification. Accordingly, the hardness decreases from 9.0 GPa to 4.8 GPa, and the Young’s modulus drops from 162.4 GPa to 106.8 GPa, revealing a notable reduction in stiffness and strength. Meanwhile, three lateral cracks extending outward from the indentation corners are visible in the single-crystal silicon. In contrast, no cracks are observed around the indentation on the modified silicon surface (Figure 4b). These results indicate that the modification process significantly enhances the material’s plasticity, enabling strain energy to be released gradually through plastic deformation rather than by instantaneous brittle fracture.

**Figure 5:**
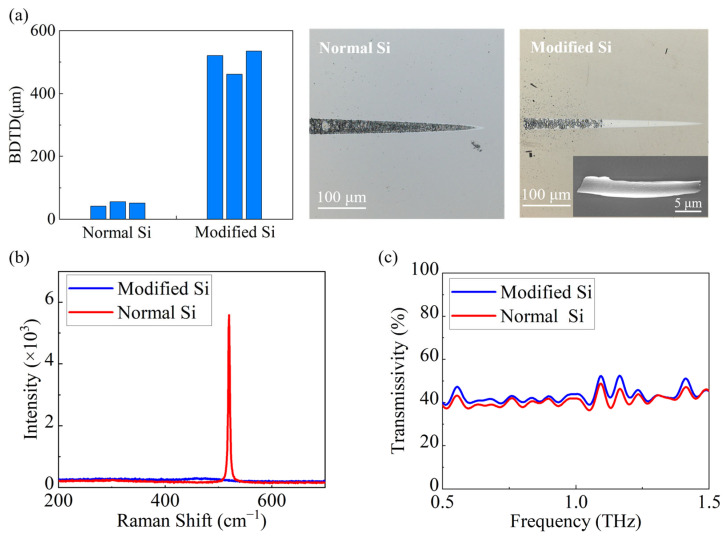
(**a**) BDTD and grooves produced by taper cutting of normal Si and modified Si; chips produced by taper cutting on the modified Si. (**b**) Raman spectra. (**c**) Terahertz transmissivity of Si before and after ion implantation.

Taper-cutting experiments also confirmed the enhanced plasticity (Figure 5a). BDTD reaches 535 nm after modification, compared with about 55 nm for unmodified single-crystal silicon. The groove morphology from taper cutting shows that the ductile removal region is substantially larger in the modified silicon. Unlike the gradual transition from ductile removal to fully brittle fracture in single-crystal silicon, the modified silicon exhibits a much sharper ductile-to-brittle transition with no apparent intermediate state, and this transition occurs at different groove depths. During taper cutting of the modified silicon, characteristic curled ductile chips are observed, with a prominent shear band in the central region, further confirming that the modification significantly improves machinability. The Raman spectrum (Figure 5b) shows that the single-crystal peak at 521 cm^−1^ disappears entirely and is replaced by a broad amorphous band centered at around 471 cm^−1^, indicating amorphization of the surface lattice. In addition, the terahertz transmission spectra of both the single-crystal and modified silicon are shown in Figure 5c. The transmittance difference between the two samples within the operating band is minimal, indicating that surface-layer amorphization has little effect on the overall transmittance. Although ion implantation may increase electrical conductivity and thus introduce additional terahertz absorption, the modified layer is only about 3 μm thick. This thickness is negligible compared with the 1 mm substrate, and therefore, terahertz propagation and transmission remain dominated by the 1 mm thick HR-Si substrate.

### 3.3. Diamond Turning of Micro-Grooves

**Figure 6:**
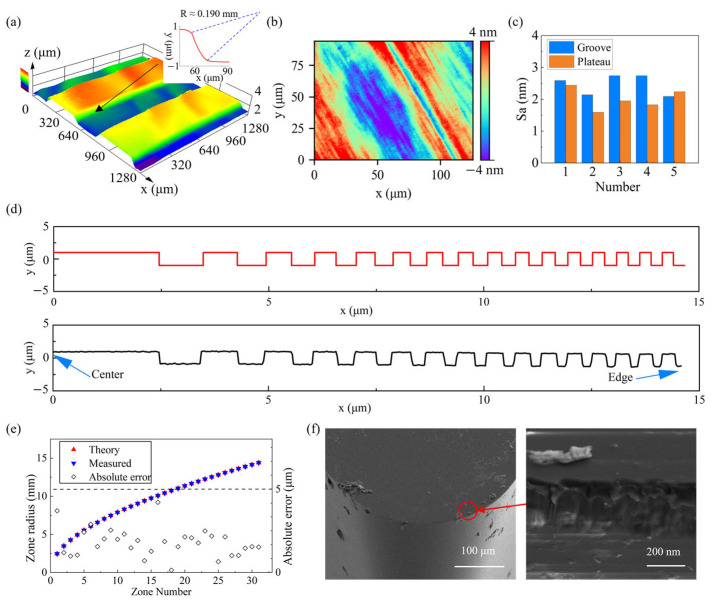
(**a**) 3D profile of Fresnel structure and groove-boundary contours. (**b**) Micro-morphology of the machined surface. (**c**) Surface roughness. (**d**) Cross-sectional profile of the Fresnel structure. (**e**) Zone radii and their absolute errors within 5 μm (dashed lines). (**f**) Morphology of the tool after machining and enlarged morphology of the cutting edge.

The partial 3D profile of the Fresnel structure after ultra-precision diamond turning is shown in Figure 6a. The groove sidewall exhibits an arc-shaped profile because the tool tip has a finite nose radius. During plunge cutting, the geometric contour of the tool nose is transferred to the groove sidewall such that the cross-sectional shape ideally approximates an arc segment with a radius equal to the tool nose radius. By fitting the measured sidewall profile, the arc radius is obtained as approximately 0.190 mm, which agrees well with the nose radius of the tool used in machining. The surface morphology after cutting is shown in Figure 6b, where the boundaries of the turning marks become less distinct, indicating a significant improvement in ductile machinability. The surface roughness Sa of the plateau and groove bottom is 1.76 ± 0.26 nm and 2.50 ± 0.33 nm, respectively, as measured by white light interferometry (Figure 6c). The surface roughness shows good uniformity within both the plateau and groove regions across the entire zone plate area. With the tool nose radius and feed kept constant, variations in the depth of cut have little influence on the theoretical roughness. However, in practical machining, the actual depth of cut in the plateau region remains within the modified layer, whereas the actual depth of cut in the groove region approaches 3 μm and thus becomes close to the boundary of the ion-implanted layer. Since amorphization near the boundary of the modified layer is often incomplete, the plastic machinability of the groove region is reduced, leading to a deterioration in surface quality compared with the plateau area after cutting. Figure 6d shows that the cross-sectional profile agrees well with the design. The cross-sectional profiles of the groove are measured, and the exact boundary positions are then identified and marked based on the extracted profiles. Subsequently, optical image stitching is performed to cover the entire radial span of the zone plate, allowing the radius of each zone boundary to be measured across the full aperture. As shown in Figure 6e, the measured radii agree well with the theoretical design, and the absolute radius error is controlled within 5 μm. Figure 6f shows an SEM image of the cutting edge after machining, where a wear land approximately 1 μm wide is observed together with chip adhesion. As mentioned above, the cutting edge operates near the boundary of the amorphous layer, and the tool is subjected to a local material response that changes with time, because amorphous, crystalline, and intermediate lattice structures may alternate as the tool moves. This induces fluctuation in the cutting force and nonuniform wear. Chip adhesion also suggests that amorphization reduces the thermal conductivity of silicon, thereby raising the temperature at the cutting edge.

### 3.4. Generation of FZP After Coating and Polishing

**Figure 7:**
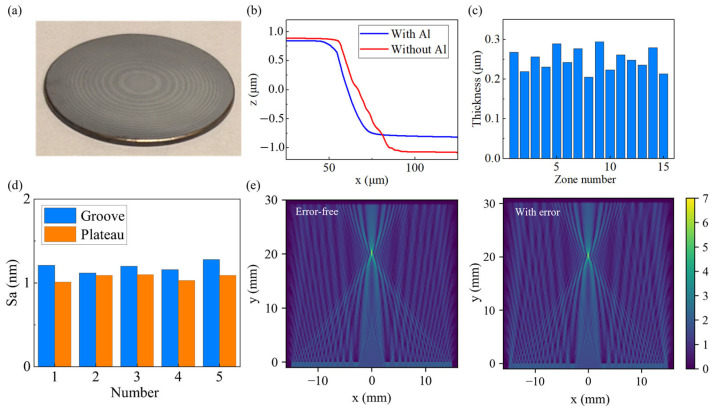
(**a**) Photograph of the FZP. (**b**) Plateau–groove boundary profiles after cutting and after polishing. (**c**) Calculated film thickness inside each groove from the center to the outermost zone. (**d**) Surface roughness of plateaus and grooves of FZP. (**e**) Electromagnetic field distributions of the theoretical and measured models.

A metal film is deposited on the microstructure surface via magnetron sputtering, forming a continuous layer across the zone-plate region without observable peeling or cracking. The deposited film accurately replicated the microstructure and preserved the micrometer-scale height difference between the groove bottoms and the plateaus after deposition. This facilitates the selective removal of the metal film from the plateaus while retaining the film inside the grooves during polishing. Based on the calibrated deposition rate and sputtering time, the film’s thickness is estimated to be around 300 nm. After polishing, the FZP is successfully fabricated (Figure 7a), and no obvious scratches are observed across the entire zone–plate area. The groove depths after diamond cutting and polishing are measured using white-light interferometry (Figure 7b). To verify the continuity of the Al film, the profiles of 15 grooves after polishing are measured along three radial directions located on the trisection lines of the zone plate in the laser mode using an Olympus confocal microscope, and their averages of three directions are shown in Figure 7c. The thickness of the remaining Al film in the grooves is estimated as the difference between the initial cutting depth (2 μm) and the post-polishing measured depth, which ranges from 200 nm to 300 nm. This variation in film thickness implies that material removal of Al may occur in some grooves during polishing. Nevertheless, the film remains thick enough to block THz waves. As shown in Figure 7d, the surface roughness Sa of the FZP is 1.15 ± 0.15 nm in the grooves and 1.04 ± 0.10 nm on the plateaus. After polishing, the residual aluminum film fills and covers the turning marks on the surface, thereby reducing the surface roughness of the plateau region. Due to the retained Al film, the roughness inside the grooves decreases compared with that of the diamond-turned Fresnel structure. An FZP model is reconstructed based on the measured dimensions, with the aluminum film thickness set to 200 nm. The electromagnetic field distribution shows no appreciable deviation from that of the theoretical model (Figure 7e), indicating that the FZP still achieves effective focusing under the present errors.

## 4. Conclusions

This study investigates a novel fabrication method for terahertz Fresnel zone plates (THz FZPs) on silicon based on the selective metallization of microgrooves. The method integrates ion implantation-assisted ultra-precision diamond turning (UPDT), magnetron sputtering coating, and polishing. The FZP structural design, material characterization, and surface measurement are conducted systematically, and the main conclusions are summarized as follows:

(1) At an operating frequency of 1 THz and a focal length of 20 mm, numerical simulations show that increasing the number of zones increases the peak focal intensity and energy fraction, suppresses out-of-focus sidelobes, and enhances focusing contrast. When the transparent-ring width error is within ±20%, the focal energy fraction and overall focusing performance remain stable.
(2) A modified layer with a thickness of about 3 μm is created on high-resistivity silicon via multi-step ion implantation, which significantly reduces the hardness and Young’s modulus and increases the ductile-to-brittle transition depth from about 55 nm to 535 nm. Machinability is enhanced, and the change in THz transmittance is negligible.
(3) Ultra-precision diamond turning produces the Fresnel structure on the modified silicon, achieving a smooth surface with a Sa roughness of 1.76–2.50 nm. Radial profile comparisons show close agreement between the machined profile and the theoretical design, with zone radius errors below 5 μm.
(4) Selective metallization is realized by depositing an Al film via magnetron sputtering, followed by polishing. The metal film on the plateau regions is removed without damaging the silicon substrate, and the film retained inside the grooves remains thicker than 200 nm, providing the required blocking functionality. The electromagnetic field distribution of the measured model shows no appreciable deviation from that of the theoretical model. These results demonstrate that the proposed method enables accurate fabrication of THz FZPs.

In future work, we will further optimize the THz setup to reduce incident wavefront distortion and perform beam profiling around the focal plane to experimentally verify the focusing performance.

## Figures and Tables

**Table 1:** Ion implantation parameters.

Number	Ions	Energy/MeV	Dosage/cm^−2^
1	Au	0.8	1 × 10^14^
2	Au	1.8	1 × 10^14^
3	Si	0.5	4 × 10^14^
4	Si	1.0	4 × 10^14^
5	Si	1.6	4 × 10^14^
6	Si	2.5	4.5 × 10^14^
7	Si	3.5	4.5 × 10^14^
8	Si	4.5	1 × 10^15^

## Data Availability

The data presented in this study are available upon request from the corresponding authors.
